# Effect of *Bacillus coagulans* DSM 32016 (TechnoSpore^®^) Supplementation on Growth Performance and Selected Blood Parameters and Serum Urea in Weaning Danube White Pigs

**DOI:** 10.3390/life16050715

**Published:** 2026-04-22

**Authors:** Katya Eneva, Gergana Yordanova, Mariyana Petrova, Radka Nedeva, Ivan Yanchev, Nikolay Karkelanov, Elena Stancheva, Toncho Penev

**Affiliations:** 1 Agricultural Institute, 9700 Shumen, Bulgaria; katiqeneva@abv.bg (K.E.); gerganaarshspb@abv.bg (G.Y.); mari_anna1305@abv.bg (M.P.); r.nedeva@abv.bg (R.N.); 2 Agricultural Academy, 1373 Sofia, Bulgaria; 3 Institute of Animal Science, 2232 Kostinbrod, Bulgaria; ijantcev@mail.bg; 4 Department of Fundamental Sciences in Animal Husbandry, Faculty of Agriculture, Trakia University, 6000 Stara Zagora, Bulgaria; niki_kyrkelanov@abv.bg; 5 Department of Ecology and Animal Hygiene, Faculty of Agriculture, Trakia University, 6000 Stara Zagora, Bulgaria; elena.stancheva@trakia-uni.bg

**Keywords:** *Bacillus coagulans* DSM 32016, probiotic supplementation, weaning pigs, serum urea, nitrogen metabolism

## Abstract

The present study evaluated the effects of dietary supplementation with *Bacillus coagulans* DSM 32016 on growth performance, hematological and biochemical parameters, and nitrogen metabolism in weaned Danube White pigs reared under standard production conditions. While supplementation did not result in statistically significant changes in average daily gain (ADG), feed conversion ratio (FCR), hematological indices, or serum lipid profile, numerical trends indicated slightly higher ADG, improved FCR, and subtle stabilization of hematological parameters in the probiotic supplemented group. Notably, serum urea concentration was significantly reduced (3.78 vs. 3.21 mmol/L; *p* = 0.017; Cohen’s *d* = 1.01), suggesting a potential positive effect on nitrogen metabolism and protein utilization efficiency. These findings are consistent with previous reports that probiotics may exert beneficial physiological effects even in the absence of statistically significant systemic changes. The observed trends highlight the potential of *Bacillus coagulans* to support growth performance and metabolic efficiency in Danube White pigs, emphasizing the importance of breed and age-specific responses in probiotic supplementation.

## 1. Introduction

The intestinal microbiota in pigs represents a dynamic ecosystem involved in the degradation and utilization of dietary substrates, as well as in the maintenance of intestinal homeostasis. Contemporary evidence emphasizes that nutritional factors can modulate the composition and metabolic activity of the gastrointestinal microbiota in swine, thereby placing the microbiome at the center of strategies aimed at optimizing productivity [1]. Interest in functional feed additives, including probiotics, has increased substantially. Probiotics are considered an alternative to antibiotic growth promoters, and their use in swine production has been the subject of systematic scientific evaluation [2]. According to available data, the effect of probiotics on productivity and health status depends on the microbial species and strain, the age of the animals, diet composition, and production conditions [3,4]. In addition to other effects, probiotics have been reported to reduce the abundance of pathogenic bacteria in the gastrointestinal tract (GIT) and to contribute to the maintenance of a stable intestinal microbiota [5,6]. Among the various probiotic microorganisms, spore-forming bacteria of the genus Bacillus are characterized by high technological stability and the ability to withstand unfavorable conditions during feed processing and passage through the gastrointestinal tract. *Bacillus coagulans* is a Gram-positive, spore-forming bacterium [7] whose potential probiotic effects in pigs have been experimentally investigated. In addition, representatives of *Bacillus* spp. exhibit tolerance to the low gastric pH, resistance to bile salts, as well as thermal stability during feed processing and long-term storage [8]. Data from piglets indicate that supplementation with *B. coagulans* may influence intestinal morphology and the expression of genes associated with barrier function, suggesting an impact on intestinal integrity and local physiology [9]. In controlled feeding trials in weaned piglets, the inclusion of *B. coagulans* in the diet has been associated with changes in growth performance and intestinal microbiota composition [10]. Furthermore, combined supplementation with *B. coagulans* has been reported to affect parameters related to immune response and intestinal barrier function [11]. The findings regarding the influence of Bacillus-based probiotics on growth performance remain inconsistent. Some studies in weaned piglets report effects on growth, digestibility, and selected blood parameters [12], whereas other investigations conducted under a wean-to-finish system indicate variable effects on productive performance depending on dietary energy density [13]. This variability underscores the need for further research across different genotypes and production settings. The strain *Bacillus coagulans* DSM 32016, used in the present study and commercially designated as TechnoSpore^®^, has been scientifically evaluated by the EFSA FEEDAP Panel, which confirmed its safety and efficacy as a feed additive for pigs [14]. Despite regulatory assessment and the availability of experimental data in piglets, published information regarding the effects of this strain on hematological and serum biochemical parameters in growing pigs remains limited. In the interpretation of blood parameters, the use of validated reference intervals for growing pigs, such as those published by Klem et al. [15], is essential in order to distinguish physiological variability from potential effects of nutritional intervention. Experimental data are available for weaned piglets but it remains unclear whether similar effects are observed in growing pigs reared under standard production conditions and in the absence of pronounced nutritional or immune stress. This developmental stage is characterized by a stabilized intestinal microbiota and relatively established metabolic homeostasis, which may modify the response to probiotic intervention. Data concerning the influence of *Bacillus coagulans* DSM 32016 on serum biochemical and hematological parameters in this age group remain limited.

The Danube White breed was developed at the Institute of Pig Breeding through a complex synthetic breeding program involving multiple founder breeds, namely Bulgarian White, Large White, Landrace, Pietrain, Hampshire, and a newly established composite genetic group. Officially recognized in 1985, the breed represents the outcome of a long-term, targeted selection strategy aimed at combining favorable genetic characteristics from the parental populations and achieving improved adaptability to local production and environmental conditions [16].

The present study aimed to evaluate the effect of supplementation with the probiotic strain *Bacillus coagulans* DSM 32016 on growth performance, feed conversion ratio, and selected blood parameters in weaning pigs.

## 2. Materials and Methods

### 2.1. Experimental Design and Animals

A total of 42 weaned pigs of the Danube White breed were included in the experiment. At the beginning of the trial, the mean body weight was 8.18 kg, increasing to 33.59 kg at the end of the 57-day experimental period. Animals were assigned to two groups (control group (CG) and experimental group (EG); *n* = 21 per group) and had ad libitum access to water. The pigs in both groups were reared under controlled environmental conditions. The microclimate in the facility was regulated by an automated electronic control system (Big Dutchman). The groups were comparable in terms of initial body weight and sex distribution (50% males and 50% females).

### 2.2. Feeding and Probiotic Supplementation

Animals in the control group were fed a standard concentrate diet. Pigs in the experimental group received the same diet supplemented with the probiotic additive TechnoSpore^®^ (Biochem, Lohne, Germany), containing the strain *Bacillus coagulans* DSM 32016, at a dose of 7 g/kg feed, delivering 1.4 × 10^11^ CFU/kg feed throughout the entire experimental period. The probiotic was incorporated into the feed using a mixer (Gruber Maschinen GmbH, Gaspoltshofen, Austria, FM 1600).

The diets provided to both groups were balanced and formulated to meet the nutritional requirements for weaned pigs. A bioconcentrate premix (Bioconcentrate mixture-12) with the following composition was used: crude protein 35.04%; crude fiber 3.65%; crude fat 6.60%; crude ash 13.92%; vitamin A 36,000 IU/kg; vitamin D_3_ 6000 IU/kg; vitamin E 450 mg/kg; total iron 695.00 mg/kg; total copper 355.00 mg/kg; lysine 1.80%; methionine 0.47%; calcium 2.60%; sodium 0.50%; phosphorus 1.20%; total zinc 390.00 mg/kg; total manganese 245.00 mg/kg; total selenium 1.45 mg/kg.

The ingredient composition as well as the energy and nutrient content of the compound feed are presented in Table 1.

### 2.3. Monitoring of Performance Parameters

During the experimental period, the following parameters were monitored:

- Feed intake—recorded daily;
- Average daily gain—calculated for the entire experimental period;
- Feed conversion ratio (feed per kg gain)—calculated for the entire period;
- Health status—monitored daily.

### 2.4. Blood Sampling and Laboratory Analyses

At the end of the experimental period, blood samples were collected from pigs in each group via the v. jugularis interna using a closed system method. Samples were collected into plastic tubes (Vacusera, Izmir, Turkey) and immediately inverted ten times. Samples intended for serum biochemistry were allowed to clot at room temperature for 2–3 h and were subsequently centrifuged at 3000× *g* for 15 min. The obtained serum was stored at −20 °C until analysis. Biochemical parameters were analyzed in extracted plasma using a BTS-350 semi-automatic biochemical analyzer (BioSystems Ltd., Barcelona, Spain). Red blood cell parameters, leukocytes, and leukocyte subpopulations were determined in whole blood using a URIT-5160 laser multidimensional hematology analyzer.

### 2.5. Statistical Analysis

The normality of data distribution was assessed using the Shapiro–Wilk test. Depending on data distribution, differences between groups were analyzed using the independent samples *t*-test or the nonparametric Mann–Whitney *U* test. The effect size for parametric analyses was calculated using Cohen’s d coefficient, determined according to the formula:

$$d=t\sqrt{\left(\frac{1}{n_{1}}+\frac{1}{n_{2}}\right)}$$

where *n*_1_ and *n*_2_ represent the number of observations in the two independent groups.

When the Mann–Whitney *U* test was applied, the effect size (*r*) was calculated according to the formula:

$$r=\frac{Z}{\sqrt{\left(N\right)}}$$

where *Z* is the standardized test statistic, and (*N = n*_1_ *+ n*_2_).

The standardized *Z* value was calculated according to the following formula:

$$Z=\frac{U-\mu U}{\sigma U}$$

where

$$\mu U=\frac{n_{1}n_{2}}{2}$$

$$\sigma U=\sqrt{\left(\frac{n_{1}n_{2}\left(n_{1}+n_{2}+1\right)}{12}\right)}$$

In these formulas, *n*_1_ and *n*_2_ represent the number of observations in the two independent groups.

The independent samples *t*-test and the Mann–Whitney *U* test were performed using GraphPad Prism (version 10.6.1, GraphPad Software Inc., La Jolla, CA, USA).

The effect of probiotic supplementation on the investigated parameters was evaluated by linear regression analysis, with treatment included as a binary independent variable (0 = control group, 1 = experimental group receiving 7 g/kg of *Bacillus coagulans* DSM 32016). The significance of the model was tested using analysis of variance (ANOVA), and regression coefficients were examined to estimate the treatment effect. Statistical analyses were conducted using JASP software (version 0.95.2). Model quality was assessed using the coefficient of determination (R^2^), which reflects the proportion of variance in the dependent variable explained by the independent variable (group), and the adjusted R^2^, which accounts for sample size and model complexity. Statistical significance was accepted at *p* < 0.05.

## 3. Results

### 3.1. Between-Group Comparisons

**Figure 1:**
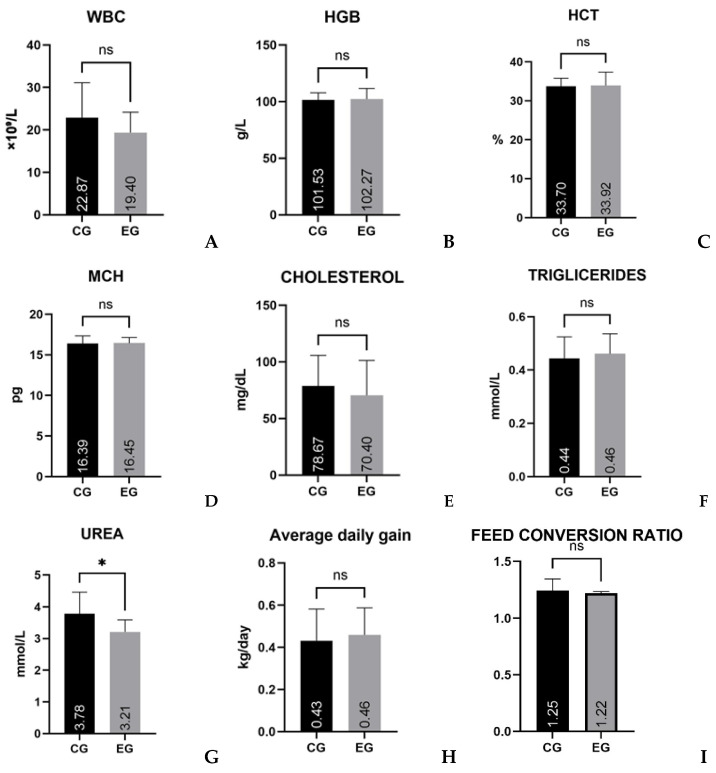
Comparison of hematological, biochemical, and performance parameters of control (CG) and experimental (EG) groups. Bars represent mean ± standard deviation. Panels (**A**–**D**) show hematological parameters (WBC, HGB, HCT, MCH); panels (**E**–**G**) show biochemical parameters (Cholesterol, Triglycerides, Urea); panels (**H**,**I**) show performance parameters (Average Daily Gain and Feed Conversion Ratio). Between-group comparisons were performed using independent samples *t*-test or Mann–Whitney *U* test according to data distribution. Statistical significance was accepted at *p* < 0.05; * indicates significant difference; ns—not significant.

The comparison between the control (CG) and experimental (EG) groups showed that differences between mean values were generally small and not statistically significant for most parameters (Table 2; Figure 1). In contrast, serum urea exhibited a more pronounced between-group effect compared to the other variables. For the hematological parameters, *t*-values ranged from 0.212 to 1.408. The highest value was observed for WBC (*t* = 1.408, *p* = 0.173), with mean values of 22.87 × 10^9^/L for CG and 19.40 × 10^9^/L for EG. Despite an absolute difference of 3.47 × 10^9^/L and a moderate effect size (Cohen’s d = 0.51), the difference was not statistically significant. For HGB (101.53 vs. 102.27 g/L), HCT (33.70% vs. 33.92%), and MCH (16.39 vs. 16.45 pg), between-group differences were minimal, as reflected by low *t*-values (0.212–0.251), high *p*-values (0.804–0.834), and negligible effect sizes (Cohen’s *d* = 0.08–0.09). As shown in Figure 1A–D, the error bars largely overlapped. Among the biochemical parameters, cholesterol showed a mean difference of 8.27 mg/dL (78.67 vs. 70.40 mg/dL), with *t* = 0.740 and *p* = 0.466, corresponding to a small effect size (Cohen’s d = 0.27).

Triglyceride concentrations differed by 0.02 mmol/L between groups (0.44 vs. 0.46 mmol/L), with the Mann–Whitney *U* test showing no statistically significant difference (*U* = 78, *p* = 0.541) and a small effect size (*r* = 0.12). For both biochemical parameters, as shown in Figure 1E-F, there was substantial overlap in the distribution of values between groups. The largest absolute and relative differences were observed for serum urea (3.78 vs. 3.21 mmol/L), corresponding to an approximately 15% lower mean value in the experimental group (EG). The *t*-test yielded *t* = 2.632, *p* = 0.017, and Cohen’s *d* = 1.01, indicating a large effect size between the group distributions. As shown in Figure 1G, the standard deviations overlap less compared with the other parameters. Average daily gain (ADG) was 0.43 kg/day in the control group (CG) and 0.46 kg/day in the experimental group (EG) (difference ≈ 0.03 kg/day; *U* = 103.5, *p* = 0.375, *r* = 0.16). Feed conversion ratio (FCR) was 1.25 in CG and 1.22 in EG (difference ≈ 0.03; *U* = 102, *p* = 0.339, *r* = 0.17). Differences between groups were not statistically significant. For both parameters (Figure 1H,I), variability between groups largely overlapped. Comparison of absolute differences, test statistics, and effect sizes indicated that urea values were higher compared to the other parameters, whereas the remaining variables showed minimal differences between groups.

Effect sizes (Cohen’s *d* or *r*) for all parameters are presented in Table 2.

The graphical representation of the between-group comparisons is shown in Figure 1, where panels (A–D) illustrate hematological parameters (WBC, HGB, HCT, MCH), panels (E–G) illustrate biochemical parameters (Cholesterol, Triglycerides, Urea), and panels (H,I) illustrate performance parameters (Average Daily Gain and Feed Conversion Ratio).

### 3.2. Linear Regression Analysis

Univariate linear regression analysis was performed with treatment (GROUP; coded as 0 = control and 1 = experimental) as a predictor of the investigated parameters (Table 3). For the hematological parameters, the coefficient of determination (R^2^) ranged from 0.004 to 0.022, indicating that less than 2.2% of the variance was explained by the model. For the biochemical parameters, R^2^ values were 0.017 for cholesterol and 0.010 for triglycerides. For the growth performance parameters, R^2^ was 0.005 for ADG and 0.046 for FCR. The adjusted R^2^ values were close to zero or negative for most parameters. In contrast, for serum urea, the model showed a moderate association (R = 0.467), with R^2^ = 0.218 and adjusted R^2^ = 0.197, indicating that approximately 21.8% of the variance was explained by group allocation. The regression coefficient (β = −0.542) was statistically significant (*t* = −3.171, *p* = 0.003), while the RMSE (0.526) reflected the residual variability of the model. The results of the regression analysis were consistent with the between-group comparisons, with serum urea being the only parameter showing a statistically significant effect of treatment (GROUP) and a comparatively higher proportion of explained variance.

The results of the linear regression analysis are presented in Table 3.

A statistically significant difference between groups was only observed for serum urea concentration, whereas no significant differences were detected for the hematological, biochemical, or growth performance parameters.

## 4. Discussion

This study evaluated the effects of *Bacillus coagulans* DSM 32016 on growth performance and selected hematological and biochemical parameters in weaning pigs reared under standard production conditions. The results showed no statistically significant changes in ADG, FCR, hematological profile, or lipid metabolism, whereas a statistically significant reduction was observed in serum urea concentration. The combination of test statistics, effect sizes, and regression analysis indicates that the effect on urea differs from that on the other parameters.

### 4.1. Performance Parameters

The difference in average daily gain (ADG) between groups was small (0.43 vs. 0.46 kg/day; difference ≈ 0.03 kg/day), with a small effect size (*r* = 0.16) and no statistical significance (*p* = 0.375). Feed conversion ratio (FCR) also showed minimal differences (1.25 vs. 1.22; difference ≈ 0.03; *r* = 0.17, *p* = 0.339). Regression analysis yielded low R^2^ values (≤0.046), indicating that group allocation explained only a minor proportion of the variance in growth parameters. Although these differences were not statistically significant, the numerical trends toward higher ADG and lower FCR suggest a potential positive effect of *Bacillus coagulans* supplementation on growth performance.

These observations are consistent with previous reports indicating that the effects of Bacillus-based probiotics on growth may be context-dependent. For instance, Jørgensen et al. [13] reported that growth-promoting effects vary depending on nutritional conditions and production phase, while Lan et al. [12] demonstrated that probiotic influence on ADG and digestibility is highly dependent on experimental context. Other studies have reported significant benefits under specific conditions [17] and showed that dietary inclusion of Bacillus based probiotics significantly increased ADG in weaned pigs. Gonzalez-Ronquillo et al. [18] found that *Bacillus* spp. supplementation in growing finishing pigs improved ADG and feed conversion.

In contrast, our study did not demonstrate statistically significant effects on growth performance, similar to the findings of Fu et al. [11], who reported more pronounced growth-promoting effects only under intestinal or immune stress conditions, which were not present in our experimental model. Similarly, Sun et al. [10] observed improved growth in younger weaned piglets, suggesting that age and physiological status may influence response to probiotics. Despite the lack of statistical significance, the numerical trends observed in ADG and FCR in the present study point to a potential beneficial effect of the probiotic, and importantly, no negative effects on productivity were detected, indicating that supplementation did not compromise growth efficiency under standard production conditions.

### 4.2. Hematological Profile

The hematological parameters WBC, HGB, HCT, and MCH did not differ significantly between groups (*p* > 0.05). Regression analysis confirmed that group allocation had negligible effects on these parameters, with very low R^2^ values (0.004–0.022), negative adjusted R^2^ values, and non-significant regression coefficients (β = −1.647 for WBC, 1.316 for HGB, 0.337 for HCT, 0.226 for MCH; *p* > 0.37). Effect sizes were negligible (*d* = 0.08–0.09) or moderate but not statistically significant (WBC, *d* = 0.51), and variability largely overlapped between groups. All values fell within the published reference intervals for growing pigs [15], indicating preserved hematological stability.

Although these differences were not statistically significant, the numerical trends observed, such as slight increases in HGB and MCH and a minor decrease in WBC, may suggest a potential beneficial effect of *Bacillus coagulans* supplementation on hematological parameters. Similarly, Fu et al. [19] reported that dietary supplementation with *Bacillus coagulans* did not lead to statistically significant changes in growth performance or hematological parameters in weaned piglets. Nevertheless, they observed positive trends in immune and antioxidant markers, including lysozyme, IL-10, C3, C4, and enhanced serum antioxidant capacity. Dlamini et al. [20] also found no significant changes in the hematological profile of clinically healthy pigs receiving probiotic supplementation. The consistency among these observations suggests that, under stable production conditions and in the absence of infectious or nutritional stress, supplementation with *Bacillus coagulans* does not produce measurable changes in classical hematological parameters but could still potentially have beneficial physiological and metabolic effects.

### 4.3. Lipid Metabolism

Serum cholesterol and triglyceride concentrations did not differ significantly between groups. Regression analysis confirmed that group allocation had negligible effects on these parameters, with very low R^2^ values (0.017 for cholesterol, 0.010 for triglycerides), negative adjusted R^2^ values, and non-significant regression coefficients (β = −6.858 for cholesterol, β = 0.014 for triglycerides; *p* > 0.4). Wu et al. [9] reported that *Bacillus coagulans* can influence intestinal morphology and barrier function in weaned piglets, which could potentially affect metabolic parameters, including the lipid profile. Similarly, Pu et al. [21] noted that combined supplementation with *Bacillus coagulans* and other functional components may modulate immune status and intestinal barrier integrity, although effects on serum lipid parameters were inconsistent. The present findings are compatible with these observations and indicate that, under stable production conditions and in the absence of intestinal stress, supplementation with *Bacillus coagulans* does not alter lipid metabolism, and the serum lipid profile remains stable.

### 4.4. Serum Urea and Nitrogen Metabolism

Serum urea concentration was significantly lower in the experimental group (3.78 vs. 3.21 mmol/L; *p* = 0.017), with a large effect size (Cohen’s *d* = 1.01). Regression analysis confirmed the significant effect of group allocation (β = −0.542; *t* = −3.171; *p* = 0.003), consistent with the between-group comparison. Urea is a key indicator of nitrogen catabolism, reflecting the balance between dietary protein intake and amino acid utilization. Bacillus-based probiotics have been described as capable of producing exogenous enzymes that enhance the degradation of dietary substrates [22], while improvements in intestinal barrier function and microbial activity following *Bacillus coagulans* supplementation have been reported by Fu et al. [11] and Pu et al. [21]. Although no direct measures of digestibility or microbial composition were performed in the present study, the observed reduction in serum urea occurring in the absence of changes in growth performance or lipid profile may indicate a potential beneficial effect of the probiotic on nitrogen metabolism. These findings suggest that *Bacillus coagulans* could improve protein utilization efficiency, highlighting a physiological effect that is detectable even under standard production conditions.

### 4.5. Scientific Contribution

The present study contributes to the existing body of evidence by demonstrating that, in clinically healthy weaning pigs reared under standard production conditions, supplementation with *Bacillus coagulans* DSM 32016 did not result in statistically significant changes in growth performance, hematological parameters, or lipid profile. Nevertheless, the observed numerical trends toward higher ADG, lower FCR, and slight improvements in hematological stability suggest potential beneficial effects on physiological resilience and nutrient utilization. Importantly, serum urea concentration was significantly reduced in the experimental group, indicating a possible positive influence on nitrogen metabolism and protein utilization efficiency. These findings provide a more nuanced understanding of probiotic effects under standard production conditions, highlighting that subtle physiological benefits may occur even in the absence of overt systemic or performance changes.

## 5. Limitations

The study was conducted within a single production cycle and under specific feeding and management conditions. Blood parameters were assessed once at the end of the experimental period. The absence of direct measures of digestibility, nitrogen balance, or intestinal microbiota limits the ability to determine the mechanism underlying the observed reduction in serum urea.

## 6. Perspectives

Future studies in Danube White pigs should include direct assessment of nitrogen balance, protein digestibility, and microbial composition to further clarify the physiological relevance of the observed reduction in serum urea. Additionally, the potential effects of probiotic supplementation on growth performance, including average daily gain (ADG), feed conversion ratio (FCR), and meat quality, should be investigated. Studies under different nutritional and physiological conditions in this breed would help define the boundaries of these effects and their potential practical significance while considering possible breed-specific responses to probiotics.

## 7. Conclusions

Supplementation with *Bacillus coagulans* DSM 32016 in weaning pigs for a period of 57 days did not result in statistically significant changes in growth performance, feed conversion ratio, hematological profile, or lipid metabolism under the conditions of the present study. Mean ADG and FCR values were higher and lower, respectively, in the experimental group, reflecting numerical trends that did not reaching statistical significance. A statistically significant reduction was only identified for serum urea, accompanied by a large effect size and confirmed by regression analysis. The observed findings suggest a possible influence on parameters related to nitrogen metabolism, which warrants further investigation through direct assessments of nitrogen balance and protein digestibility.

## Figures and Tables

**Table 1:** A. Ingredient composition (%). B. Calculated nutrient composition (per kg feed). Ingredient composition and calculated nutrient content of the basal diet (as-fed basis).

**A.**	
Ingredient	%
Wheat	43.0
Barley	25.0
Protein–vitamin–mineral concentrate	32.0
Total	100.0
**B.**	
Parameter	Value
Digestible energy, kcal	3168
Metabolizable energy, kcal	3032
Crude protein, %	17.8
Lysine, g	0.82
Crude fiber, %	3.66
Crude fat, %	3.25
Nitrogen-free extract (NFE) *	556
Calcium, g	0.95
Phosphorus, g	0.60

* NFE—as provided in the original feed formulation data.

**Table 2:** Effect sizes and statistical comparison between control (CG) and experimental (EG) groups.

Parameter	Test	Statistic	*df*/*N*	*p*-Value	Effect Size	Interpretation
WBC	*t*-test	*t* = 1.408	*df* = 22.49	0.173	Cohen’s *d* = 0.51	moderate effect
HGB	*t*-test	*t* = 0.2511	*df* = 24.37	0.804	Cohen’s *d* = 0.09	negligible effect size
HCT	*t*-test	*t* = 0.2119	*df* = 23.06	0.834	Cohen’s *d* = 0.08	negligible effect size
MCH	*t*-test	*t* = 0.2198	*df* = 25.60	0.828	Cohen’s *d* = 0.08	negligible effect size
Cholesterol	*t*-test	*t* = 0.7399	*df* = 24.93	0.466	Cohen’s *d* = 0.27	small effect size
Triglycerides	Mann–Whitney *U*	*U* = 78	*N* = 42	0.541	*r* = 0.12	small effect size
Urea	*t*-test	*t* = 2.632	*df* = 18.38	0.017	Cohen’s *d* = 1.01	large effect size
ADG (Average Daily Gain)	Mann–Whitney *U*	*U* = 103.5	*N* = 42	0.375	*r* = 0.16	small effect size
Feed Conversion Ratio	Mann–Whitney *U*	*U* = 102	*N* = 42	0.339	*r* = 0.17	small effect size

Notes: *df* = degrees of freedom; *N* = total number of observations; Cohen’s *d* = effect size for parametric *t*-tests, calculated as difference between group means divided by pooled standard deviation; *r* = effect size for Mann–Whitney *U* test, *t*-test = independent samples *t*-test; Mann–Whitney *U* = nonparametric test for two independent groups. Effect size interpretation: negligible (<0.2), small (0.2–0.5), moderate (0.5–0.8), large (>0.8). ADG = Average Daily Gain; WBC = White Blood Cells; HGB = Hemoglobin; HCT = Hematocrit; MCH = Mean Corpuscular Hemoglobin.

**Table 3:** Linear regression analysis of the effect of binary treatment (control vs. experimental) on hematological, biochemical, and growth parameters in pigs.

Variable	Model	R	R^2^	Adjusted R^2^	RMSE	Coefficient (β)	*t*	*p*
WBC	M_1_ (GROUP)	0.128	0.017	−0.011	6.530	−1.647	−0.777	0.442
HGB	M_1_ (GROUP)	0.084	0.007	−0.021	8.050	1.316	0.504	0.617
HCT	M_1_ (GROUP)	0.060	0.004	−0.024	2.901	0.337	0.358	0.722
MCH	M_1_ (GROUP)	0.150	0.022	−0.005	0.769	0.226	0.908	0.370
Cholesterol	M_1_ (GROUP)	0.131	0.017	−0.010	26.73	−6.858	−0.791	0.434
Triglycerides	M_1_ (GROUP)	0.102	0.010	−0.017	0.071	0.014	0.617	0.514
Urea	M_1_ (GROUP)	0.467	0.218	0.197	0.526	−0.542	−3.171	0.003
ADG (Average Daily Gain)	M_1_ (GROUP)	0.074	0.005	−0.022	163.1	23.56	0.445	0.659
FCR (Feed Conversion Ratio)	M_1_ (GROUP)	0.214	0.046	0.019	69.70	−29.68	−1.313	0.198

Model M_1_—linear regression with GROUP (control vs. experimental) as predictor; R—multiple correlation coefficient; R^2^—proportion of variance explained by the model; Adjusted R^2^—variance explained adjusted for number of predictors; RMSE—root mean square error; Coefficient (β)—effect of GROUP on the dependent variable; *t*—*t*-statistic for the coefficient; *p*—*p*-value indicating significance.

## Data Availability

The data presented in this study are available on request from the corresponding author due to restrictions related to privacy and ethical considerations.

## References

[1] Liao S.F., Ji F., Fan P., Denryter K. (2024). Swine gastrointestinal microbiota and the effects of dietary amino acids on its composition and metabolism. Int. J. Mol. Sci..

[2] Barba-Vidal E., Martín-Orúe S.M., Castillejos L. (2019). Practical aspects of the use of probiotics in pig production: A review. Livest. Sci..

[3] Rybarczyk A., Bogusławska-Wąs E., Łupkowska A. (2020). Effect of EM® probiotic on gut microbiota, growth performance, carcass and meat quality of pigs. Livest. Sci..

[4] Zimmermann J.A., Fusari M.L., Rössler E., Blajman J.E., Romero-Scharpen A., Astesana D.M., Olivero C.R., Berisvil A.P., Signorini M.L., Zbrun M.V. (2016). Effects of probiotics in swine growth performance: A meta-analysis of randomized controlled trials. Anim. Feed Sci. Technol..

[5] Yirga H. (2015). The use of probiotics in animal nutrition. J. Prob. Health.

[6] Dumitru M., Habeanu M., Lefter N.A., Gheorghe A. (2020). The effect of *Bacillus licheniformis* as direct-fed microbial product on growth performance, gastrointestinal disorders and microflora population in weaning piglets. Rom. Biotechnol. Lett..

[7] Kim K., He Y., Xiong X., Ehrlich A., Li X., Raybould H., Liu Y. (2019). Dietary supplementation of *Bacillus subtilis* influenced intestinal health of weaned pigs experimentally infected with a pathogenic *E. coli*. J. Anim. Sci. Biotechnol..

[8] Ragul K., Syiem I., Sundar K., Shetty P.H. (2017). Characterization of probiotic potential of Bacillus species isolated from a traditional brine pickle. J. Food Sci. Technol..

[9] Wu T., Zhang Y., Lv Y., Li P., Yi D., Wang L., Zhao D., Chen H.B., Gong J.S., Hou Y.Q. (2018). Beneficial impact and molecular mechanism of *Bacillus coagulans* on piglets intestine. Int. J. Mol. Sci..

[10] Sun T., Miao H., Zhang C., Wang Y., Liu S., Jiao P., Li W., Li Y., Huang Z. (2022). Effect of dietary *Bacillus coagulans* on the performance and intestinal microbiota of weaned piglets. Animal.

[11] Fu R., Liang C., Chen D., Yan H., Tian G., Zheng P., He J., Yu J., Mao X., Huang Z. (2021). Effects of dietary *Bacillus coagulans* and yeast hydrolysate supplementation on growth performance, immune response and intestinal barrier function in weaned piglets. J. Anim. Physiol. Anim. Nutr..

[12] Lan R., Tran H., Kim I.H. (2017). Effects of probiotic supplementation in different nutrient density diets on growth performance, nutrient digestibility, blood profiles, fecal microflora and noxious gas emission in weaning pigs. J. Sci. Food Agric..

[13] Jørgensen J.N., Laguna J.S., Millán C., Casabuena O., Gracia M.I. (2016). Effects of a Bacillus-based probiotic and dietary energy content on the performance and nutrient digestibility of wean-to-finish pigs. Anim. Feed Sci. Technol..

[14] EFSA FEEDAP Panel (2020). Safety and efficacy of TechnoSpore® (*Bacillus coagulans* DSM 32016) for pigs. EFSA J..

[15] Klem T.B., Bleken E., Morberg H., Thoresen S.I., Framstad T. (2010). Hematologic and biochemical reference intervals for Norwegian crossbreed grower pigs. Vet. Clin. Pathol..

[16] Slanev S., Stojkov A., Petrov P. (2006). Efficient Pig Farming.

[17] Mun D., Kyoung H., Kong M., Ryu S., Jang K.B., Baek J., Park K.I., Song M., Kim Y. (2021). Effects of Bacillus-based probiotics on growth performance, nutrient digestibility, and intestinal health of weaned pigs. J. Anim. Sci. Technol..

[18] Gonzalez-Ronquillo M., Villegas-Estrada D., Robles-Jimenez L.E., Garcia Herrera R.A., Villegas-Vázquez V.L., Vargas-Bello-Pérez E. (2022). Effect of the Inclusion of *Bacillus* spp. in Growing-Finishing Pigs’ Diets: A Meta-Analysis. Animals.

[19] Fu R., Chen D., Tian G., Zheng P., Mao X., Yu J., He J., Huang Z., Luo Y., Yu B. (2019). Effect of dietary supplementation of *Bacillus coagulans* or yeast hydrolysates on growth performance, antioxidant activity, cytokines and intestinal microflora of growing-finishing pigs. Anim. Nutr..

[20] Dlamini Z.C., Langa R.L., Aiyegoro O.A., Okoh A.I. (2017). Effects of probiotics on growth performance, blood parameters and gastrointestinal microbial population of weaned pigs. S. Afr. J. Anim. Sci..

[21] Pu J.N., Chen D.W., Tian G., He J., Zheng P., Mao X.B., Yu J., Huang Z.Q., Luo J.Q., Luo Y.H. (2020). Effects of benzoic acid, *Bacillus coagulans* and oregano oil combined supplementation on growth performance, immune status and intestinal barrier integrity of weaned piglets. Anim. Nutr..

[22] Gu S.B., Zhao L.N., Wu Y., Li S.C., Sun J.R., Huang J.F., Li D.D. (2015). Potential probiotic attributes of a new strain of *Bacillus coagulans* CGMCC 9951 isolated from healthy piglet feces. World J. Microbiol. Biotechnol..

